# Relationship between nurses' perceptions of the benefits/challenges of nursing and degree of interprofessional and intraprofessional collaboration in all‐inclusive services combining day services, overnight stays and home‐visit nursing for the older people living at home

**DOI:** 10.1002/jgf2.655

**Published:** 2023-10-30

**Authors:** Nobuko Katahira, Satomi Maruo

**Affiliations:** ^1^ Department of Nursing Chiba Prefectual University of Health Sciences Chiba Japan; ^2^ Graduate School of Health and Welfare Sciences International University of Health and Welfare Graduate School Tokyo Japan; ^3^ Kobe City College of Nursing Hyogo Japan

**Keywords:** aging in place, elderly, interprofessional collaboration, multifunctional long‐term care in small group homes and home‐visit nursing, nursing

## Abstract

**Background:**

Many countries are experiencing rapid population aging, and the provision of support for older adults with diseases or disabilities to continue living in their communities is a major global challenge. Japan has established multifunctional long‐term care in small group homes and home‐visit nursing (MLSH) as a service category that integrates medical and care services. These services focus on nursing functions to support continuous, long‐term home, and end‐of‐life care for older adults with high levels of medical care dependency. This study aimed to clarify the relationship between nurses' perceptions of nursing benefits/challenges and the degree of interprofessional collaboration in the context of MLSH.

**Methods:**

We conducted a mail questionnaire survey of MLSH facilities throughout Japan. All facilities in Japan that had been operating for at least 1 year were included. We analyzed 182 responses (response rate: 36.0%; valid response rate: 98.3%).

**Results:**

Comparison of scores representing the degree of interprofessional collaboration perceived by nurses showed the highest score was for colleague nurses (3.9 ± 0.5) and the lowest was for external care managers (2.5 ± 0.9). Compared with the weak collaboration group, the strong collaboration group had higher perceptions of the benefits of nursing and lower perceptions of the challenges.

**Conclusions:**

The results of this study suggest that strong collaboration allows teams to achieve sufficient effects of care while reducing related challenges. It may be necessary to promote collaboration with external professionals to appropriately manage service users' worsened conditions and improve the quality of care.

## 1 INTRODUCTION

Many countries are experiencing rapid population aging.^1^ Home care for older people has been promoted to improve conventional medical and welfare systems and reduce medical costs in countries with rapidly aging populations. For example, various care programs have been adopted in the United States, including the Program of All‐inclusive Care for the Elderly (PACE)^2^ and the Community Aging in Place, Advancing Better Living for Elders (CAPABLE) program.^3^ The PACE^2^ is a comprehensive, community‐based model of care that coordinates medical, behavioral, and social services for individuals 55 and above who have high care needs but can safely remain in the community. A previous study showed the PACE was associated with reduced hospitalizations and increased mental and physical health among patients, and caregiver stress was alleviated. Similarly, the CAPABLE program^3^ was shown to contribute to facility users' health. However, little is known about how PACE outcomes compare with those of similar caregiving programs internationally. In addition, there are few published comparisons of PACE outcomes versus those of other caregiving programs for older adults in the United States, and available studies reported mixed results.^4^

In Japan, older people account for 29.0% of the total population.^5^ The Long‐term Care Insurance system plays a central role in the welfare of this population group.^6^ Japan provides multifunctional long‐term care in small group homes and home‐visit nursing (MLSH) via a “community‐based service category,” which integrates medical and welfare services for community‐dwelling older people. This community‐based service category was established when the Long‐term Care Insurance system was revised in April 2012. Multifunctional small group homes (MSGH) comprise another service category, which is mainly centered on day services combined with overnight stays and home visits to support the home lives of older people that require care on a 24‐hour basis. Both MSGH and MLSH facilities limit the number of users to 29 people. Professionals employed by these facilities include care managers, nurses, and care workers. MSGH services have only 1.5 nurses on average,^7^ which limits the provision of nursing services. MLSH is an extended version of MSGH that includes home‐visit nursing. Compared with MSGH, MLSH has enhanced nursing functions, meaning MLSH facilities can provide support and end‐of‐life care for older people with high levels of medical care dependency. Various nursing benefits that characterize MLSH services have been reported, including enabling users to continue their home lives (even in difficult cases) and expanding the scope of care through interprofessional and intraprofessional cooperation.^8^ However, some challenges have also been noted, such as inappropriate acceptance of or care for users, and difficulty educating and cooperating with care workers^8^; these areas require improvement.

In the primary care context, interprofessional collaboration has been reported to improve pain care.^9^ In addition, patient‐centered interdisciplinary interventions appear to be more effective than usual care.^10^ Nursing and care services in MLSH are flexibly combined and provided according to the condition and needs of individual users. Collaboration among MLSH nurses and external physicians and care managers is also required, with interprofessional and intraprofessional collaboration being likely to become increasingly important. Therefore, we aimed to examine whether the benefits and challenges of nursing in MLSH varied according to the degree of collaboration between MLSH nurses and other professionals. Clarifying the relationships between these nursing benefits/challenges and interprofessional and intraprofessional collaboration may help in developing measures to improve or promote good relationships and open discussion about effective nursing approaches in MLSH. We anticipate that, if they demonstrate the effectiveness of nursing care in MLSH and identify the factors associated with effective nursing care, the results of this study will help nurses to support patient discharge. This may also contribute to the development of effective support strategies for older people living at home in other aging societies.

## 2 OBJECTIVE

The aim of this study was to clarify the relationships between MLSH nurses' perceptions of the benefits/challenges of nursing and degree of interprofessional and intraprofessional collaboration.

## 3 METHODS

### 3.1 Study design

This study used a cross‐sectional design.

### 3.2 Participants

Although the number of MLSH facilities is increasing, it remains small. Therefore, we decided to include all facilities that had been active for at least 1 year. Participants were managers and nursing directors of 514 MLSH facilities registered with the Care Service Information Publication System in Japan.^11^

### 3.3 Data collection date

October to December 2019.

### 3.4 Procedure

We conducted this self‐administered questionnaire survey via mail. The survey form, an invitation letter, and a stamped self‐addressed envelope were mailed to potential participants.

### 3.5 Study items

Our survey collected information on facility managers, facilities, and service users (e.g., type of facility, number of employees, service users' age, and care grade) and nurses' attributes (e.g., gender, age, and length of practical experience in MLSH). We measured interprofessional and intraprofessional collaboration using the Relational Coordination Scale (RCS),^12^ which was developed to measure teamwork with specific subjects about specific tasks.

A previous study that used the RCS revealed that professionals' satisfaction with the care delivered by community health nurses was positively influenced by relational coordination and enhanced relational coordination between community health nurses and other primary care professionals in the neighborhood, which may improve the delivery of care to community‐dwelling frail people. This study used the Japanese version of the RCS (J‐RCS^13^;). Respondents were asked to rate seven profession‐specific items on a five‐point Likert scale (from “Always/Quite often” to “Never/Seldom”). Relational coordination items encompass four communication dimensions (frequent, timely, accurate, and problem‐solving communication) and three relationship dimensions (shared goals, shared knowledge, and mutual respect). The sum of the scores for these seven questions divided by 7 gives a total score that represents the ease of collaborating with each profession. Higher scores indicated that nurses perceived good coordination with other team members. The Cronbach's alpha coefficient for the RCS was 0.86–0.90 in a study of nurse managers.^14^ Cronbach's alpha for the J‐RCS was 0.78–0.86 in a study of home care nurses.^13^ This scale has previously been shown to have sufficient reliability and validity^13^ and has been used by other studies in the community nursing field. One study found that home‐visiting nurses' relational coordination with their nursing managers was significantly and negatively related to depersonalization.^15^ Another study that used the RCS showed that enhanced relational coordination between community health nurses and other neighborhood primary care professionals may improve the delivery of care to community‐dwelling frail people.^16^ Furthermore, relational coordination was reported to be positively associated with higher job satisfaction, better work engagement, lower burnout, lower turnover, and reciprocal learning among healthcare professionals.^17^ Therefore, we considered that the RCS was a suitable scale for measuring interprofessional and intraprofessional collaboration in the community, and used this scale in our study.

We measured respondents' perceptions of 33 items covering the benefits/challenges of MLSH nursing. Responses were on a 5‐point Likert scale ranging from “Strongly agree” to “Strongly disagree” (“Strongly agree” corresponded to a rating of “5” on the Likert scale). These benefits and challenges were identified through the results of our previous survey of MLSH nurses in the Kansai area,^8^ and an additional survey in another area. We created a draft benefits/challenges questionnaire after adding items and modifying expressions based on the feedback received from those nurses. We then asked two MLSH nurses to test the draft, and reviewed each question based on their feedback. Finally, we developed a 33‐item questionnaire comprising 14 benefits and 19 challenges.

We asked two nursing directors to pretest the draft questionnaire we had developed. On the basis of the pretest results, we modified the expressions of some of the 33 items on the advantages/problems of MLSH nursing, but made no changes to the J‐RCS.

### 3.6 Analysis

The degree of collaboration with each profession was determined by the median J‐RCS score. Using this score, we divided respondents from each occupation into two groups: a strong coordination group and a weak coordination group. In a previous study, nurses were divided into two groups according to the mean score,^7^ but in this study, we considered it appropriate to divide participants into two groups using the median because the normality of the distribution could not be assumed. We compared the two groups using Mann–Whitney *U*‐tests. The level of statistical significance was set at *p* < 0.05.

### 3.7 Ethical considerations

This survey included an invitation letter that specified the study objective, measures to ensure participants' anonymity, voluntary participation, and the consent process. Respondents were advised that returning a completed survey would be considered provision of consent to participate in this study. The completed anonymous questionnaires were filed and stored in a locked vault. Electronic data were analyzed and stored only on password‐controlled PCs managed by the research institution, and both paper and electronic data were managed only by the first author (the principal investigator). This study was approved by the Ethics Committee of Chiba Prefectural University of Health Sciences (No.2019‐21).

## 4 RESULTS

### 4.1 Outline of facilities, service users, and nurses

Of the 185 responses received (response rate: 36.0%), we analyzed 182 valid responses (valid response rate: 98.3%). The most common facility type was for‐profit corporations (*n* = 80; 44.0%), and 110 (60.4%) facilities had an adjoining home‐visit nursing facility. Among the nursing directors who responded, 163 (90.1%) were female, 64 (35.4%) were aged 40–49 years, 168 (92.8%) were full‐time employees, and 83 (45.6%) were concurrently managers. Among the service users registered in a given month, there were 6.9 ± 3.9 males and 13.7 ± 5.2 females, and the most common age group was 80–89 years (9.8 ± 3.7 users), followed by 90–99 years (5.4 ± 3.2 users). The most common care grades were 5 (4.4 ± 3.0 users) and 4 (4.4 ± 2.2 users). In total, 3.1 ± 3.8 users had received end‐of‐life care in the facility over the past 1 year (Table 1). Furthermore, among all facility users, 8.7 ± 7.3 lived alone (including those living alone only during the daytime), and 8.0 ± 6.9 regularly received home‐visit medical services.

**TABLE 1:** Outline of facilities, users, and nurses.

		Number of people	(%)
Facilities			
Type of facility	Profit corporation	80	44
	Medical facility	43	23.6
	Social welfare facility	35	19.2
	Others	21	11.5
	No response	3	1.6
Users			
Average number of registered users/month	Male	6.9 ± 3.9	
	Female	13.7 ± 5.2	
Age, years	≥90s	5.4 ± 3.2	
	80s	9.8 ± 3.7	
	70s	4.0 ± 2.4	
	60s	1.2 ± 1.2	
Long‐term care grade^a^	Care grade 5	4.4 ± 3.0	
	Care grad e4	4.4 ± 2.2	
	Care grade 3	4.0 ± 2.2	
	Care grade 2	4.4 ± 2.7	
	Care grade 1	3.6 ± 2.8	
Nurses			
Gender	Female	163	90.1
	Male	18	9.9
Age, years	60s	34	18.8
	50s	58	32
	40s	64	35.4
	≤30s	25	13.8
Employment	Full‐time	168	92.8
	Part‐time	13	7.2
Length of practical experience in MLSH (years)		2.6 ± 1.7	

*Note*: MLSH, multifunctional long‐term care in small group homes and home‐visit nursing. Ministry of Health, Labour and Welfare.^18^

^a^ The long‐term care grade is an index based on the Long‐term Care Insurance system that indicates the level of need for care services. Those classified as care grades 1, 2, and 3 are permitted to use insurance‐covered care services for 32–49, 50–69, and 70–89 min per day, respectively.

### 4.2 Relationships between J‐RCS scores and nursing benefits/challenges

The highest J‐RCS score (range: 0–5) was for colleague nurses (3.9 ± 0.5), followed by colleague care managers (3.8 ± 0.5) and colleague care workers (3.6 ± 0.6). Compared with these professional groups, scores for physicians in charge and external care managers were lower at 3.1 ± 0.7 and 2.5 ± 0.9, respectively (Table 2).

**TABLE 2:** J‐RCS score for each profession; mean ± SD (*N* = 182).

	Colleague nurses	Colleague care workers	Colleague care managers	External care managers	Physicians
	3.9 ± 0.5	3.6 ± 0.6	3.8 ± 0.5	2.5 ± 0.9	3.1 ± 0.7
Reference: Evaluation of the home‐visit nursing care station^a^	(4.2 ± 0.4)	–	(3.8 ± 0.6)	–	(3.1 ± 0.6)

Abbreviations: J‐RCS: Japanese version of the Relational Coordination Scale; SD, standard deviation.

^a^ Naruse et al.^13^

After confirming that there were no ceiling effects in the scores for each profession, we compared the level of perceived nursing benefits/challenges between the strong and weak collaboration groups. When limited to items with significant differences, the strong collaboration group showed higher perceptions of nursing benefits and lower perceptions of challenges compared with the weak collaboration group. This tendency was observed for 23 of the 33 items when comparing strong (J‐RCS score ≥ 4, *n* = 97) and weak (J‐RCS score < 4, *n* = 84) collaboration with colleague nurses. Similar trends with significant differences were observed in 22 items for colleague care workers, 19 items for colleague care managers, and 13 items for external care managers and physicians.

For external care managers, there were significant differences according to the degree of collaboration in three benefits, and the weak collaboration group had significantly higher perceptions of 10 of the 19 challenges than the strong collaboration group. For physicians, there were significant differences between the two collaboration groups in 10 of the 14 benefits and three of the 19 challenges (Tables 3 and 4).

**TABLE 3:** Relationships between nurses' perceptions of the nursing benefits and degree of interprofessional collaboration (*N* = 182).

	Colleague nurses			Colleague care workers			Colleague care managers			External care managers			Physicians		
	Strong	Weak	*p*‐Value	Strong	Weak	*p*‐Value	Strong	Weak	*p*‐Value	Strong	Weak	*p*‐Value	Strong	Weak	*p*‐Value
	*n* = 97	*n* = 84		*n* = 60	*n* = 121		*n* = 61	*n* = 120		*n* = 39	*n* = 142		*n* = 113	*n* = 68	
1. Allowing nurses to implement elaborate care approaches from their perspective	4.1 ± 0.7	3.5 ± 0.8	<0.001	4.1 ± 0.7	3.6 ± 0.8	<0.001	4.0 ± 0.8	3.7 ± 0.8	0.024						
2. Allowing symptom management that is difficult for care workers to perform	4.4 ± 0.6	3.8 ± 0.8	<0.001	4.4 ± 0.6	3.9 ± 0.8	<0.001	4.3 ± 0.7	4.0 ± 0.8	0.012						
3. Empowering nurses	3.7 ± 0.9	3.1 ± 1.1	<0.001	3.7 ± 1.0	3.3 ± 1.0	0.004	3.7 ± 0.9	3.3 ± 1.0	0.014	3.7 ± 0.8	3.4 ± 1.0	0.021	3.7 ± 0.9	3.1 ± 1.1	0.000
4. Helping clarify conditions at home/night and adjust medications	4.0 ± 0.8	3.3 ± 0.8	<0.001	4.0 ± 0.8	3.5 ± 0.9	<0.001	3.9 ± 0.9	3.5 ± 0.9	0.007				3.8 ± 0.8	3.4 ± 0.9	0.001
5. Helping clarify conditions at home/night and identify symptoms	3.9 ± 0.8	3.3 ± 0.8	<0.001	4.0 ± 0.8	3.5 ± 0.8	0.000	3.9 ± 0.8	3.5 ± 0.9	0.009	3.9 ± 0.8	3.6 ± 0.9	0.037	3.8 ± 0.8	3.3 ± 0.8	<0.001
6. Preventing accidents, even during sudden overnight stays, based on users' diurnal conditions	3.9 ± 0.9	3.4 ± 0.9	0.000	4.0 ± 0.8	3.5 ± 0.9	0.001	3.9 ± 1.0	3.5 ± 0.8	0.015						
7. Enabling users to continue their home lives, even in difficult cases	3.9 ± 0.9	3.5 ± 1.0	0.003	3.9 ± 0.9	3.6 ± 0.9	0.040							3.9 ± 0.8	3.4 ± 1.0	0.001
8. Preventing hospitalization, even in users who have repeatedly been hospitalized and discharged	3.8 ± 0.9	3.3 ± 0.9	0.000	3.9 ± 0.9	3.4 ± 0.9	0.002	3.8 ± 0.9	3.4 ± 0.9	0.004				3.8 ± 0.9	3.2 ± 0.9	<0.001
9. Making it possible to manage worsened conditions within the facility without hospitalization	3.6 ± 1.1	3.2 ± 1.0	0.004				3.7 ± 1.1	3.3 ± 1.0	0.025	3.8 ± 1.0	3.4 ± 1.1	0.029	3.7 ± 1.0	3.0 ± 1.1	<0.001
10. Allowing flexible care plan changes according to users'/families' situations	4.1 ± 0.8	3.7 ± 0.9	0.000	4.2 ± 0.8	3.8 ± 0.8	0.004							4.0 ± 0.8	3.7 ± 0.9	0.011
11. Helping fulfill users'/families' desires	4.0 ± 0.8	3.7 ± 0.7	0.003	4.1 ± 0.8	3.8 ± 0.7	0.044							4.0 ± 0.8	3.7 ± 0.7	0.003
12. Connecting to home services and making it possible to support families	4.0 ± 0.8	3.5 ± 0.9	<0.001	4.1 ± 0.8	3.6 ± 0.9	<0.001	4.0 ± 0.8	3.6 ± 0.9	0.006				3.9 ± 0.9	3.5 ± 0.8	0.005
13. Expanding the scope of care by promoting cooperation between nurses and care workers	4.0 ± 0.8	3.4 ± 0.8	<0.001	4.3 ± 0.7	3.5 ± 0.8	<0.001	4.1 ± 0.8	3.6 ± 0.9	<0.001						
14. Making it possible to provide care from various perspectives through multiprofessional approaches	4.0 ± 0.7	3.4 ± 0.8	<0.001	4.2 ± 0.7	3.4 ± 0.8	<0.001	3.9 ± 0.8	3.6 ± 0.8	0.003				3.8 ± 0.8	3.5 ± 0.9	0.008

*Note*: Only values for items with significant differences are shown. Active collaboration group: median J‐RCS scores: colleague nurses/colleague care workers/colleague care managers ≥4; external care managers/physicians ≥3.

**TABLE 4:** Relationships between nurses' perceptions of nursing challenges and degree of interprofessional collaboration (*N* = 182).

	Colleague nurses			Colleague care workers			Colleague care managers			External care managers			Physicians		
	Strong	Weak	*p*‐Value	Strong	Weak	*p*‐Value	Strong	Weak	*p*‐Value	Strong	Weak	*p*‐Value	Strong	Weak	*p*‐Value
	*n* = 97	*n* = 84		*n* = 60	*n* = 121		*n* = 61	*n* = 120		*n* = 39	*n* = 142		*n* = 113	*n* = 68	
15. Difficulty providing nursing care in the absence of a physician										2.4 ± 0.9	3.0 ± 1.1	0.005			
16. Difficulty educating nurses working different hours	2.7 ± 1.1	3.1 ± 1.0	0.012	2.6 ± 1.0	3.0 ± 1.1	0.019									
17. Difficulty educating nurses with different experience	2.8 ± 1.1	3.3 ± 1.0	0.004	2.8 ± 1.0	3.2 ± 1.1	0.014	2.7 ± 1.0	3.2 ± 1.1	0.004	2.7 ± 1.0	3.1 ± 1.1	0.018			
18. Difficulty of cooperation among nurses because of differences in attitudes	2.4 ± 1.0	3.2 ± 0.9	<0.001	2.4 ± 1.0	3.0 ± 1.0	0.000	2.5 ± 1.0	2.9 ± 1.0	0.030						
19. Heavy burden on nurses who manage an increased number of users and also provide care															
20. Difficulty in accepting elderly people with moderate to severe conditions that are occasionally difficult to manage	2.5 ± 1.2	2.9 ± 1.0	0.016				2.4 ± 1.1	2.8 ± 1.1	0.020	2.4 ± 1.0	2.8 ± 1.1	0.042	2.5 ± 1.1	3.0 ± 1.0	0.007
21. Users referred by home care support providers, who are all difficult cases															
22. Exhaustion among staff because of continuous overnight stays															
23. Difficulty providing individualized care for users	2.3 ± 0.9	2.8 ± 1.0	<0.001	2.3 ± 1.0	2.7 ± 1.0	0.008				2.2 ± 0.9	2.6 ± 1.0	0.022			
24. Difficulty providing end‐of‐life care with families' understanding	2.2 ± 1.1	2.6 ± 1.0	0.010							2.1 ± 0.9	2.4 ± 1.1	0.030	2.2 ± 1.0	2.6 ± 1.1	0.011
25. Difficulty obtaining families' understanding of service use							2.2 ± 0.9	2.6 ± 1.0	0.002	2.1 ± 1.0	2.6 ± 1.0	0.004			
26. Difficulty educating care workers with different education backgrounds				2.6 ± 1.0	3.4 ± 1.0	<0.001	2.8 ± 1.1	3.3 ± 1.0	0.009	2.7 ± 1.1	3.2 ± 1.1	0.007			
27. Difficulty cooperating with other nurses who have different ideas	2.6 ± 1.2	3.0 ± 1.0	0.002	2.1 ± 1.0	3.1 ± 1.1	<0.001	2.4 ± 1.2	2.9 ± 1.0	0.011	2.4 ± 1.1	2.8 ± 1.2	0.039			
28. Tendency of care workers to excessively rely on nurses in care				2.2 ± 1.2	3.0 ± 1.2	<0.001									
29. Difficulty collaborating with physicians in terms of communication							2.2 ± 0.8	2.5 ± 1.0	0.032	2.1 ± 0.9	2.5 ± 1.0	0.016	2.2 ± 0.9	2.8 ± 1.0	<0.001
30. Difficulty collaborating with colleague care managers	1.7 ± 1.0	2.2 ± 1.0	0.001	1.6 ± 1.0	2.1 ± 1.0	0.001	1.6 ± 1.0	2.2 ± 1.0	0.000	1.5 ± 0.7	2.0 ± 1.0	0.002			
31. Insufficient recognition of MLSH and its capabilities															
32. Difficulty providing sufficient care for users who are unable to use other services	2.4 ± 1.1	2.8 ± 1.0	0.015	2.3 ± 1.0	2.7 ± 1.1	0.027									
33. The current system that does not allow people with lower care grades to use these services															

*Note*: Only values for items with significant differences are shown. Active collaboration group: median J‐RCS scores: colleague nurses/colleague care workers/colleague care managers ≥4; external care managers/physicians ≥3.

Abbreviations: J‐RCS, the Japanese version of the Relational Coordination Scale; MLSH, multifunctional long‐term care in small group homes and home‐visit nursing.

With regard to the relationship between nursing benefits and profession, the strong collaboration group for all professions had significantly higher scores than the weak collaboration group for two items: “empowering nurses” (colleague nurses: 3.7 ± 0.9 vs. 3.1 ± 1.1, *p* < 0.001; colleague care workers: 3.7 ± 1.0 vs. 3.3 ± 1.0, *p* = 0.004; colleague care managers: 3.7 ± 0.9 vs. 3.3 ± 1.0, *p* = 0.014; external care managers: 3.7 ± 0.8 vs. 3.4 ± 1.0, *p* = 0.021; physicians: 3.7 ± 0.9 vs. 3.1 ± 1.1, *p* = 0.000) and “helping clarify conditions at home/night and identify symptoms” (3.9 ± 0.8 vs. 3.3 ± 0.8, *p* < 0.001; 3.9 ± 0.8 vs. 3.3 ± 0.8, *p* < 0.001; 3.9 ± 0.8 vs. 3.5 ± 0.9, *p* = 0.009; 3.9 ± 0.8 vs. 3.6 ± 0.9, *p* = 0.037; 3.8 ± 0.8 vs. 3.3 ± 0.8, *p* < 0.001, for those professions respectively).

In terms of the relationships between nursing challenges and professions, the level of perceived “difficulty in providing nursing care in the absence of a physician” was significantly lower among those who collaborated strongly with external care managers than in the weak collaboration group (2.4 ± 0.9 vs. 3.0 ± 1.1, *p* = 0.005). The level of perceived “tendency of care workers to excessively rely on nurses in care” was also significantly lower among those who collaborated strongly with colleague care workers than in the weak collaboration group (2.2 ± 1.2 vs. 3.0 ± 1.2, *p* < 0.001).

## 5 DISCUSSION

Our examination of the relationships between nursing benefits/challenges and interprofessional and intraprofessional collaboration showed that the level of perceived benefits tended to be higher and that of challenges tended to be lower in the strong collaboration group compared with the weak collaboration group. This suggested that teams in which members (regardless of their profession) favorably communicated with each other and shared goals could achieve sufficient effects of care while reducing related challenges. These results supported those of previous studies that showed that interprofessional collaboration improved the quality of care for users.^9^, ^10^ A previous study showed that psychological safety strengthened the relationships among hospital nurses.^19^ In this study, some nurses perceived there to be lower “Difficulty of cooperation among nurses because of differences in attitudes” when cooperation among nurses was stronger, which suggested that psychological safety among nurses strengthened cooperation and decreased the perceived difficulty in cooperation. For issues that tended to be regarded as challenges in nurse–nurse relationships (represented by “difficulty in educating nurses with different experiences”), the level of perceived challenges was relatively low among those that collaborated strongly with colleague care workers and care managers. Another noteworthy aspect of the findings was that the level of perceived nursing challenges in MLSH tended to be low among external care managers in the strong collaboration group. In the current system, MLSH nurses rarely exchange user information directly with external care managers, as shown by the small number of external care managers with strong collaboration in this study. Furthermore, the perceived level of “empowering nurses” (as a benefit) was higher in the strong collaboration group for all professions. These findings suggested that interprofessional and intraprofessional collaboration may be essential to address nursing issues and improve the quality of nursing.

The results of this study indicated that collaboration with external professionals was difficult; therefore, the degree of collaboration perceived by nurses remained low. A previous study identified hierarchy and authoritarian leadership as barriers to psychological safety in multidisciplinary teams in the primary care context.^20^ Hierarchy and authoritarian leadership may also be factors associated with nursing challenges, such as “difficulty in collaborating with physicians in terms of communication.” However, although the hierarchical culture in the healthcare context negatively affects communication, industry–academia collaboration promotes communication.^21^ Therefore, cooperation with research institutions may also be an effective strategy to improve communication.

The results of the present study suggest that strong interprofessional and intraprofessional collaboration promoted the benefits of nursing and helped to resolve challenges. Both education^22^ and use of communication tools^23^ are considered effective in promoting collaboration. Therefore, we propose that multiple professions receive training together, including training with outside professionals, on the theme of strengthening interprofessional cooperation and the use of communication tools. Furthermore, in Japan's Long‐term Care Insurance system, additional fees for the improvement of care worker management may serve as an incentive for training care workers or creating training opportunities to improve their quality of care. It is likely that the medical fee system will be revised to apply this category to nursing, meaning more training seminars on municipal subsidies and use of communication tools could be held going forward. To improve cooperation between professions, it would also be useful to raise awareness and increase managerial support in using daily duties as opportunities to strengthen cooperation. For example, an MLSH nurse could be present at regular medical visits to ask family physicians about the health of particular clients that the staff were concerned about, and to obtain instructions from the physician. In this context, nurses can act as mediators between the staff and the attending physician. Therefore, managers should try to coordinate nurses' work schedules to facilitate such mediation and thereby encourage interprofessional collaboration.

Various countries appear to be developing systems in various forms to comprehensively support the daily lives of older people. For example, in the United States, the “Comprehensive Primary Care Initiative,” which was a primary care practice transformation model, was found to reduce hospitalizations and emergency department visits.^24^ In addition, a Canadian initiative, “Seniors” Campus Continuums', offers a model of care that seeks to broaden access to an array of services and housing options to meet the growing health and social needs of aging populations.^25^ MLSH is an example of this type of comprehensive support system. One outcome of the present study was that we identified interprofessional and intraprofessional collaboration as an important factor in improving the quality of care in MLSH. In addition, we highlighted the importance of promoting collaboration, both within a facility and with external professionals. These findings may be useful to inform the development and review of systems for home care based on cooperation among multiple professionals.

## 6 LIMITATIONS

This study has a number of limitations. For example, it may be difficult to generalize our results because of the low response rate (36.0%). In addition, we could not determine causal relationships between nursing activities and study factors because this study used a cross‐sectional design. Furthermore, this study only included nurses in management roles. The perceived benefits and disadvantages related to collaboration may differ between nurse managers and nurses actually working in the field. Although our study clarified the relationship between the strength of interprofessional and intraprofessional collaboration and the perception of nursing benefits/challenges, we were not able to show the actual effects of nursing on facility users. We believe that a further study is necessary to verify these effects based on our results. There are also challenges for nurses associated with working with physicians, and it may be effective to start with a shared vision and provide appropriate training on communication techniques and tools with the help of research institutions.

Our study suggested that multidisciplinary training is necessary. In addition, it is desirable to review the public system and consider subsidizing local governments to provide incentives for nursing staff to participate in training focused on multidisciplinary cooperation.

## 7 CONCLUSION

We conducted a national survey to clarify the relationship between nursing benefits/challenges and interprofessional and intraprofessional collaboration in the context of all‐inclusive services that combine day services, overnight stays, and home‐visit nursing for older people living at home. Nurses in the group with strong interprofessional collaboration perceived higher nursing benefits and lower challenges than those with weak collaboration. Teams that had strong collaboration perceived more nursing benefits, as well as fewer challenges. It is therefore essential to strengthen interprofessional and intraprofessional collaboration to improve the quality of nursing care and support nurses in coping with the increasing severity of their patients.

However, as the degree of collaboration with external professionals in general was relatively low, it may be important to promote collaboration with external physicians and care managers to ensure appropriate management of worsened mental and physical conditions among facility users and improve the quality of care. Because nurses experience challenges in collaborating with physicians, we recommend that training be provided in communication techniques and tools, starting with a shared vision. Relevant research institutions could assist with such training efforts. To improve collaboration, it would be useful to increase awareness of the importance of using daily duties as an opportunity to strengthen collaboration between professions, and to increase support from managers. Finally, our results suggest multidisciplinary training is necessary, and it may be appropriate to review the public system and offer subsidies for local governments to provide incentives for nursing staff to participate in training on multidisciplinary cooperation.

## AUTHOR CONTRIBUTIONS

NK corresponded about the scale used in this study and contributed to data collection, data entry, and writing of the manuscript. SM provided valuable support throughout the study and contributed to data analysis, writing, and critical review of the manuscript.

## CONFLICT OF INTEREST STATEMENT

The authors declare no conflicts of interest.

## References

[1] United Nations . World Population Prospects 2019: Highlights. 2019. Available from https://population.un.org/wpp. Accessed Feb 17, 2021

[2] Gyurmey T , Kwiatkowski J . Program of all‐inclusive Care for the Elderly (PACE): integrating health and social care since 1973. R I Med J. 2019;102(5):30–32. 31167525

[3] Fields B , Yanes C , Ennis M , Toto P . Community aging in place, advancing better living for elders (CAPABLE) program: understanding the potential involvement of care partners. Health Soc Care Community. 2022;30(4):e1212–e1219. 10.1111/hsc.13529 34355833

[4] Arku D , Felix M , Warholak T , Axon DR . Program of all‐inclusive Care for the Elderly (PACE) versus other programs: a scoping review of health outcomes. Geriatrics. 2022;7(2):31. 10.3390/geriatrics7020031 35314603 PMC8938794

[5] Ministry of Internal Affairs and Communications . Current Population Estimates as of October 1, 2022. 2022. Available from https://www.stat.go.jp/english/data/jinsui/2022np/index.html. Accessed May 24, 2022

[6] Ministry of Health, Labour and Welfare . Long‐Term Care Insurance System of Japan. 2016. Available from https://www.mhlw.go.jp/english/policy/care‐welfare/care‐welfare‐elderly/dl/ltcisj_e.pdf Accessed May 24, 2023

[7] Katahira N , Tsukasaki K . Nursing care in multifunctional small group homes‐providing day, visiting, and overnight services for older people living at home. Int J Nurs Pract. 2016;22(6):605–615. 10.1111/ijn.12482 27653637

[8] Katahira N , Ogawa T , Maruo S . Benefits and challenges of nursing in multifunctional long‐term care in a small group home and home‐visit nursing, and ideas for nursing: an analysis of nurses' perceptions. J Gen Fam Med. 2020;43(2):54–61 (in Japanese). 10.14442/generalist.43.54

[9] Campwala Z , Davis G , Khazen O , Trowbridge R , Nabage M , Bagchi R , et al. The impact of multidisciplinary conferences on healthcare utilization in chronic pain patients. Front Pain Res. 2021;22(2):775210. 10.3389/fpain.2021.775210 PMC8915707 35295478

[10] Lamper C , Beckers L , Kroese M , Verbunt J , Huijnen I . Interdisciplinary care networks in rehabilitation Care for Patients with chronic musculoskeletal pain: a systematic review. J Clin Med. 2021;10(9):2041. 10.3390/jcm10092041 34068727 PMC8126257

[11] Ministry of Health, Labour and Welfare . Public Notice of Long‐Term Care Service Information System. n.d. Available from https://www.kaigokensaku.mhlw.go.jp (in Japanese). Accessed May 24, 2023

[12] Gittell JH , Fairfield KM , Bierbaum B , Head W , Jackson R , Kelly M , et al. Impact of relational coordination on quality of care, postoperative pain and functioning, and length of stay: a nine‐hospital study of surgical patients. Med Care. 2000;38(8):807–819. 10.1097/00005650-200008000-00005 10929993

[13] Naruse T , Sakai M , Nagata T . Reliability and validity of the Japanese version of the relational coordination scale. Jpn J Public Health. 2014;61(9):565–573. 10.11236/jph.61.9_565 25298090

[14] Warshawsky NE , Havens DS , Knafl G . The influence of interpersonal relationships on nurse Managers' work engagement and proactive work behavior. J Nurs Adm. 2012;42(9):418–425. 10.1097/NNA.0b013e3182668129 22922751 PMC4071766

[15] Cao X , Naruse T . Effect of time pressure on the burnout of home‐visiting nurses: the moderating role of relational coordination with nursing managers. Jpn J Nurs Sci. 2019;16(2):221–231. 10.1111/jjns.12233 30203464

[16] Cramm JM , Hoeijmakers M , Nieboer AP . Relational coordination between community health nurses and other professionals in delivering care to community‐dwelling frail people. J Nurs Manag. 2014;22:170–176. 10.1111/jonm.12041 23441966

[17] House S , Wilmoth M , Kitzmiller R . Relational coordination and staff outcomes among healthcare professionals: a scoping review. J Interprof Care. 2021;36(6):891–899. 10.1080/13561820.2021.1965101 34392784

[18] Ministry of Health, Labour and Welfare . The Long‐Term Care Grading System. 2018. Available from http://www.mhlw.go.jp/topics/kaigo/nintei/gaiyo2.html (in Japanese). Accessed May 24, 2023

[19] Ahlstedt C , Eriksson LC , Holmström IK , Muntlin ÅN . Flourishing at work: Nurses' motivation through daily communication – an ethnographic approach. Nurs Health Sci. 2020;22(4):1169–1176. 10.1111/nhs.12789 33104296 PMC7756815

[20] Remtulla R , Hagana A , Houbby N , Ruparell K , Aojula N , Menon A , et al. Exploring the barriers and facilitators of psychological safety in primary care teams: a qualitative study. BMC Health Serv Res. 2021;21(1):269. 10.1186/s12913-021-06232-7 33761958 PMC7988250

[21] LaMothe J , Hendricks S , Halstead J , Taylor J , Lee E , Pike C , et al. Developing interprofessional collaborative practice competencies in rural primary health care teams. Nurs Outlook. 2020;S0029–6554(20):30708–30709. 10.1016/j.outlook.2020.12.001 33386146

[22] Congdon HB , Gina C , Rowe C , Pittman J , Goodwin J . Teaching interprofessional practice skills to nursing, social work and pharmacy students in primary care health centers. J Interprofessional Educ Pract. 2020;21:100373. 10.1016/j.xjep.2020.100373

[23] Coolen E , Engbers R , Draaisma J , Heinen M , Fluit C . The use of SBAR as a structured communication tool in the pediatric non‐acute care setting: bridge or barrier for interprofessional collaboration? J Interprof Care. 2020;Nov 15:1–10. 10.1080/13561820.2020.1816936 33190546

[24] Fu N , Singh P , Dale S , Orzol S , Peikes D , Ghosh A , et al. Long‐term effects of the comprehensive primary care model on health care spending and utilization. J Gen Intern Med. 2022;37(7):1713–1721. 10.1007/s11606-021-06952-w 34236603 PMC9130381

[25] Morton‐Chang F , Majumder S , Berta W . Seniors' campus continuums: local solutions for broad spectrum seniors care. BMC Geriatr. 2021;21(1):70. 10.1186/s12877-020-01781-8 33472583 PMC7816473

